# Avirulence genes of *Magnaporthe oryzae* are a pathogen defense arsenal shaped by cognate rice resistance genes in the field

**DOI:** 10.1111/nph.71586

**Published:** 2026-10-12

**Authors:** Xueyan Wang, Grant T. Billings, Amanda M. Hulse‐Kemp, Quentin D. Read, Yulin Jia, Melissa H. Jia, Yixiao Huang, Yeshi Wamishe, Barbara Valent

**Affiliations:** ^1^ Rice Research and Extension Center University of Arkansas Stuttgart AR 72160 USA; ^2^ Dale Bumpers National Rice Research Center USDA, ARS Stuttgart 72160 AR USA; ^3^ Crop and Soil Sciences Department North Carolina State University Raleigh NC 27695 USA; ^4^ Genomics and Bioinformatics Research Unit USDA, ARS Raleigh NC 27695 USA; ^5^ Southeast Area, USDA, ARS Raleigh NC 27606 USA; ^6^ Kansas State University Manhattan KS 66506 USA

**Keywords:** avirulence gene, evolutionary arms race, generalized linear modeling, longitudinal pathogen survey, *Oryza sativa*, resistance gene, rice blast fungus

## Abstract

- We examined the frequency of and variation in eight avirulence (*AVR*) genes from 840 *Magnaporthe oryzae* field isolates collected from the Southern United States relative to rice resistance (*R*) genes deployed from 1959 to 2017. Little coding or noncoding variation was detected in *AVR‐Pi9*, *AVR‐Pib*, *ACE1* and *AVR‐Pizt*, which correspond to *R* genes not used in the United States.
- In the United States, *AVR‐Pik*, corresponding to rice *R* gene *Pi‐k* alleles deployed at varying levels since the late 1960s, was present in 37% of the isolates over six decades. *AVR‐Pita1*, corresponding to *Pi‐ta* deployed in the late 1990s, reached a low point in the 5‐yr period following *Pi‐ta* deployment and then recovered to 99% in recent years.
- Our results suggest a fitness penalty for *M. oryzae* isolates carrying nonfunctional AVR variants and provide concrete data on how changes in plant *R* gene composition can impact pathogen populations. These findings will inform localized strategies for *R* gene usage and durable control of blast disease.
- *AVR* frequencies differ over time between United States and global samples for *AVR‐Pik*, *AVR‐Pi9*, *AVR‐Pii* and *AVR‐Pita1*. Further whole genome analysis including additional US samples is necessary to confirm whether the United States is a geographically isolated population.

## Graphical abstract

Population structure and *AVR* gene presence/absence of 383 single‐spore isolates from the US *Mo* population.

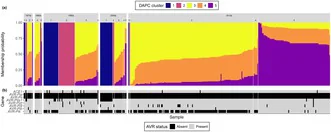

## Introduction

Rice (*Oryza sativa* L.) is a major staple crop that feeds over half of the world population. Blast disease of rice caused by the fungus *Magnaporthe oryzae* (T.T. Hebert) M.E. Barr (synonym of *Pyricularia oryzae* Cavara) is one of the foremost constraints to secure stable rice production. *Magnaporthe oryzae* (*Mo*) is a highly adaptable pathogen with a genome containing abundant mobile elements, and some strains contain dispensable supernumerary chromosomes (Dean *et al*., 2005; Peng *et al*., 2019). In the United States in the 2020s, blast in rice is managed using an integrated approach: the use of host resistance (*R*) genes, which are then coupled with fungicides and cultural practices such as management of paddy flood level. In the United States, where only 1–2% of global rice is grown, 70 million USD is estimated to be required annually in the mid‐south alone to suppress the disease (Nalley *et al*., 2016). Globally, a loss of 66 billion USD is estimated due to blast, equivalent to an amount that is enough to feed 60 million people (Pennisi, 2010). The use of *R* genes in rice is the most economic and sustainable method to manage blast disease. To date, nearly 100 major *R* genes have been identified from cultivated and wild rice relatives, of which 50+ have been molecularly characterized world‐wide (B. H. Wang *et al*., 2017; Zhao *et al*., 2018; Kou *et al*., 2024). In the United States, *R* genes, *Pi‐*a, *Pi‐b*, *pi‐d, Pi‐i, Pi‐km/Pi‐ks*, *Pi‐ta* and *Pi‐z*, have been deployed (X. Wang *et al*., 2017).

The major blast *R* genes in rice and corresponding avirulence (*AVR*) genes in the fungus follow a ‘gene‐for‐gene’ interaction, in which the effectiveness of any *R* gene depends on the expression of the corresponding *AVR* alleles encoding the recognized pathogen effector (Silué *et al*., 1992). The presence of an *R* gene in a host imposes a fitness cost on the pathogen's corresponding *AVR* genes by arresting infection (Vera Cruz *et al*., 2000; Leach *et al*., 2001; Figueroa *et al*., 2016). In the absence of the associated *R* gene, a fitness penalty (reduced aggressiveness and lack of persistence) for the pathogen could lead to increased durability for that *R* gene (Vera Cruz *et al*., 2000; Leach *et al*., 2001). For example, the rice *Xa7* gene confers resistance to the bacterial blight pathogen *Xanthomonas oryzae*, through a gene‐for‐gene interaction with the *AVR* gene *AvrXa7*. It has been shown that *X. oryzae* mutants lacking AVR activity are less aggressive in both laboratory and field studies, and they fail to persist in field populations, suggesting that *Xa7* should provide good disease control over time through a fitness penalty mechanism (Vera Cruz *et al*., 2000). In the rice–*M. oryzae* pathosystem, deletion of entire *AVR* genes is a common mechanism for the pathogen to evade host *R* genes. However, analysis of *AVR* gene deletion mutants has failed to identify loss of aggressiveness phenotypes or candidates for durable *R* genes in this rice‐blast pathosystem. Deletion of *AVR* genes has facilitated the cloning processes of *AVR‐Pita1*, *AVR‐Pia*, *AVR‐Pii* and *AVR‐Pik/km/kp* (Orbach *et al*., 2000; Miki *et al*., 2009; Yoshida *et al*., 2009).

To date, 12 *AVR* genes including *PWL2*, *AVR1‐CO39*, *AVR‐Pita*, *ACE1*, *AVR‐Pia*, *AVR‐Pii*, *AVR‐Pik*, *AVR‐Pizt*, *AVR‐Pi9*, *AVR‐Pib*, *PWT3* and *PWT4* in *M. oryzae* have been characterized from pathogen isolates originating in rice and nonrice hosts (Orbach *et al*., 2000; Yoshida *et al*., 2009; Kang *et al*., 1995; Sweigard *et al*., 1995; Farman & Leong, 1998; Fudal *et al*., 2005; Li *et al*., 2009; Ribot *et al*., 2013; Wu *et al*., 2015; Zhang *et al*., 2015; Inoue *et al*., 2017).

*AVR‐Pita* encodes a predicted metalloprotease that directly recognizes the product of cognate *R* gene *Pi‐ta* (Jia *et al*., 2000). The function of *Pi‐ta* requires the *Ptr* gene, an armadillo type *R* gene. Together, these genes constitute the highly resistant gene cluster *Pita/Pi42*/*Ptr* (Zhao *et al*., 2018; Meng *et al*., 2020). *AVR‐Pita* belongs to a small gene family and was renamed *AVR‐Pita1* (Khang *et al*., 2008). The DNA sequences of *AVR‐Pita1* alleles are highly diverse, with previous reports indicating high nucleotide diversity in the coding regions (Huang *et al*., 2014; Singh *et al*., 2014). All functional *AVR‐Pita1* haplotypes were identified from avirulent isolates, and they genetically interact with the only known functional *Pi‐ta* allele. Deletion of large DNA fragments or insertion of transposable elements within *AVR‐Pita1* in these isolates causes a gain in virulence activity (Zhou *et al*., 2007; Dai *et al*., 2010; Xing *et al*., 2013; Singh *et al*., 2014).

*Pi‐km* recognizes *AVR‐Pik‐D, ‐E* and ‐*A* alleles (Kanzaki *et al*., 2012) as well as the structurally unrelated effector *AVR‐Mgk1* (Sugihara *et al*., 2023). By contrast, *Pi‐ks* recognizes only *AVR‐Mgk1* and does not recognize any *AVR‐Pik* alleles; therefore, AVR‐Mgk1 is the cognate effector for *Pi*‐*ks*. All instances of ‘*AVR‐Pik* alleles’ in this manuscript refer to *AVR‐Pik* alleles and not *AVR‐Mgk1*. *AVR‐Pik/km/kp* encodes a predicted MAX effector (de Guillen *et al*., 2015). The *AVR‐Pik* haplotypes determined resistance specificity toward *R* alleles, *Pi‐km, Pi‐kh* and *Pi‐kp* at the *Pi‐k* locus (Yoshida *et al*., 2009; Kanzaki *et al*., 2012; Wu *et al*., 2014; Li *et al*., 2019). Complete deletion of the *AVR‐Pik* gene causes rice cultivars with cognate *R* genes to become susceptible (Yoshida *et al*., 2009). Loss of *AVR*‐*Pia* and *AVR‐Pii* also alter host disease resistance to susceptibility (Miki *et al*., 2009; Yoshida *et al*., 2009).

Rice blast *AVR* gene dynamics determine the efficiencies of the *R* genes deployed in rice fields. Many snapshots of *AVR* gene composition have documented the complexities and variation between pathogen populations at different locations (Li *et al*., 2014; Selisana *et al*., 2017; Xing *et al*., 2017). Two independent studies using 131 isolates (Lopez *et al*., 2019) and 77 isolates from different rice growing areas in the Philippines (Selisana *et al*., 2017) reported presence frequencies of 81.50% vs 21.9% for *AVR‐Pik*, 64.16% vs 0% for *AVR‐Pita*, 47.98% vs 84.9% for *AVR‐Pii*, 19.08% vs 2.7% for *AVR‐Pizt* and 5.20% vs 0% for *AVR‐Pia*, respectively. Zhang *et al*. rapidly analyzed *AVR‐Pia*, *AVR‐Pii*, *AVR‐Pizt* and *AVR‐Pik* in 232 isolates from 1975 to 2009 using PCR (Zhang *et al*., 2020). Because *AVR* gene frequencies can differ between locations, it is crucial to understand the dynamic pathogen populations. Additionally, pathogen *AVR* repertoire information should be sufficient to predict *R* gene efficacy for that region at that point in time. Longitudinal data are needed to model and project *R* gene durability in response to changing *M. oryzae AVR* genes across locations over time.

We previously reported that analysis of the US *Mo* population with neutral markers showed remarkable diversity relative to world‐wide diversity. In our previous work, most variation was apparent over time rather than across geographic regions, although *Mo* is under asexual reproduction in the field (X. Wang *et al*., 2017). Here, we tried to test our expectations from the gene‐for‐gene hypothesis, that is rice host resistance is a major driving force for genetic variability in the blast fungus. We directly test this hypothesis by characterizing eight *AVR* genes in 840 US field isolates collected from 1959 to 2017. We analyzed historical rice production records for this same time period in order to understand the relationship of the *AVR* genes with their cognate *R* genes in the field. Little variation, either in *AVR* gene presence or in protein haplotype variation, occurred for four *AVR* genes lacking cognate *R* genes in the field. Two *AVR* genes corresponding to *R* genes widely deployed in the late 1960s and 1970s have been rarely detected in the US pathogen population since then. Among the *AVR* genes examined in this study, only *AVR‐Pita1* and *AVR‐Pik* had diversity in amino acid sequences, with 37 and seven variants, respectively. *AVR‐Pita1* gene frequency recovered to high levels after removal of *Pi‐ta* from the field, with rare isolation of the single pathotype lacking AVR activity. *AVR‐Pik* gene frequency also increased after frequency of *Pi‐km* declined. The findings in the changes of *AVR* genes correlated with the deployed cognate *R* genes over six decades suggesting the *AVR* genes in *Mo* are an evolving defense arsenal. This knowledge is useful for developing rice varieties with the desired resistance to control blast disease. Understanding regional *AVR* gene dynamics adds valuable information for predicting and deploying durable resistance to blast disease.

## Materials and Methods

### Fungal materials and detection and DNA sequencing of the *AVR* genes

A total of 840 field isolates of *Magnaporthe oryzae* (T.T. Hebert) M.E. Barr were collected from 1959 to 2017 across the US rice (*Oryza sativa* L.) production areas (AR, TX, LA, FL, MS, MO – Supporting Information Table S1). Due to low sample numbers, samples from FL were grouped with TX, and MO/TN were grouped with AR. Samples from before 1974 were excluded from the change overtime analysis due to very low sample numbers, although they were included in evolutionary analyses. The archived *M. oryzae* isolates before 2000 were collected by Dr. Toni Marchetti (ARS, Beaumont, TX, USA). From 2000 onwards, different individuals collected the isolates. Details of the purification, isolation and storage of *M. oryzae* samples is provided in Methods S1. Details of DNA isolation and PCR are provided in Methods S1.

For *AVR* gene detection (Table S1), several sets of *AVR* gene specific primers were used to ensure that the absence of amplification was not due to nonstructural, sequence variation at primer sites (Table S2; Methods S3). Except for *AVR‐Pia and AVR‐Pii*, all *AVR* gene‐specific primers were used for DNA sequencing of the entire open reading frame (ORF), plus 5′ and 3′ untranslated regions of the *AVR* genes. The polymerase chain reaction (PCR) products were purified by ExoSAP methods and Sanger sequenced at USDA‐ARS Genomics and Bioinformatics Research Unit (Stoneville, MS, USA). Specific fluorescence primers for *AVR‐Pia* were designed using Primer‐Blast (Ye *et al*., 2012; Table S2), and specific fluorescence primers for *AVR‐Pii* were designed by Zhang *et al*. (2020; Table S2) for rapid detection. DNA of *M. oryzae* in Dataset S1 was extracted following a protocol described by Jia *et al*. (2014) with modifications that follow. Briefly, a piece of filter paper containing blast mycelia was put in a 0.2‐ml tube with 100 μl lysis buffer (100 mM Tris–HCl, 2 mM EDTA, pH 8.0). The sample was heated at 95°C for 10 min and centrifuged at 850 ***g*** for 1 min. The solution was then diluted 1 : 3 with Tris‐EDTA (TE) buffer. PCR was conducted as described previously (B. H. Wang *et al*., 2017; X. Wang *et al*., 2017). Annealing temperatures used for *AVR‐Pia* and *AVR‐Pii* were 67°C and 55°C, respectively.

*AVR* genes were detected in publicly available resequencing datasets (see ‘DNA sequence analysis’ section for further detail) using a primer sequence match counting approach (Dataset S2). The ‘locate’ subcommand in seqkit v2.9.0 was used to count the instances of exact matches to each of the fast‐detection primer pairs in the quality‐trimmed fastq files. An *AVR* gene was declared ‘present’ if both primer sequences were observed at least five times in a library and absent otherwise.

Changes in the *AVR* gene frequency were plotted using the R package ggplot2 v3.5.2 (Wickham, 2016). Smooth predictions of the *AVR* gene frequency by year, along with the SE for the point‐in‐time estimates, on the response (probability) scale were generated with a cubic spline using the ‘gam’ and ‘predict.gam’ functions in the R package mgcv v1.9‐1 (Wood, 2011). Pathogen *AVR* gene detections were grouped by year and treated as a binomial response in a generalized linear model (details follow).

Details of testing of isolates for pathogenicity against rice varieties is provided in Methods S4.

### *AVR* and *R* gene frequency analyses

The acreages of 160 rice varieties in four states (AR, TX, LA and MS) along with their *R* genes were collected from available documentary sources (Tables S3, S4). Due to sparsity problems (no data for AR 1960–1963; AR, LA, MS or TX for 1974; LA, MS and TX for 1977–1989 and 2000; MS for 2014), values were interpolated before statistical modeling (Dataset S2). Varieties not listed in a particular year for a state were set to zero acres. For LA, MS and TX, acreages of each rice variety were copied forward from 1976 to 1977–1981 and copied backward from 1990 to 1985–1989. Then, the ‘approx’ function, which uses linear interpolation, in R v4.4.3 was used for linear interpolation for all years having missing acreage.

The *R* gene's frequency in each state and year was estimated using the interpolated rice acreage values along with the R gene presence/absence status collected from the literature. The *R* gene frequency was calculated using the following formula:

$$P\left(R_{j}|\text{State},\text{Year}\right)=\frac{\sum_{i=1}^{160}A_{\text{State},\text{Year}}\left(i\right)\times I_{R_{j}}\left(i\right)}{\sum_{i=1}^{160}A_{\text{State},\text{Year}}\left(i\right)\times \left(I_{R_{j}}\left(i\right)+I_{R_{\neg j}}\left(i\right)\right)}$$

where *P*(*R*_*j*_|State,Year) is the probability of a randomly sampled field or plot to contain a resistance gene *j* in a specific state and year. $I_{R_{j}}$ is an indicator variable that takes value 1 if the rice variety is known to contain the *R* gene *j*, and 0 otherwise. $I_{S_{j}}$ is an indicator variable that takes value 1 if the rice variety is known *not* to contain the *R* gene *j*, and 0 otherwise. Rice varieties with unknown *R* gene status were entirely excluded (i.e. did not contribute to the denominator) from this analysis.

We hypothesized that *R* genes could impact the frequency of *AVR* genes in the field in a lagged fashion. For this reason, we first did some model selection for each *AVR* gene and their corresponding *R* genes to determine which continuous time interval in prior years to use to predict *AVR* gene frequencies. A time‐series lag was constructed by averaging the *R* gene frequencies in each state, starting from *N* years back and going up to *U* years before the current year. For instance, if *n* = 5 and *U* = 3 and the focal year was 2000, then the *R* gene frequencies for 1995, 1996 and 1997 were averaged to form the new predictor. The lagged time series that had the lowest *P*‐value for the linear term in the following generalized linear mixed model was selected for the next stage of modeling:

$$\text{logit}\left(p_{{\mathrm{AVR}}_{j,k,l}}\right)=1+S_{k}+\beta \times p\left(R_{j,\mathrm{kl}}\right)$$

where the response is the log‐odds of the proportion of samples containing a given *AVR* gene *j* in state *k* with lagged *l R*‐gene proportion *R* in state *k*. The state *S* was treated as a random effect because we want to generalize about what could happen in new localities if *R‐*gene frequencies were manipulated there as well. A single slope *β* was modeled because we expect the *AVR* gene frequencies to respond the same way to changes in *R*‐gene frequencies regardless of locality. An uninformative Gaussian prior, with mean 0 and SD 5 on the log‐odds scale, was set on the slope term to ensure model convergence. The global intercept captures the predicted *AVR* gene frequency in an average state with no *R*‐gene present.

The model was fit using the R package glmmTMB v1.1.11.9000 (Brooks *et al*., 2017). Model predictions for changes in *AVR* gene frequency in response to changes in *R* gene frequency were made using the ‘predict’ function, and visualized on the response (proportion, not log‐odds) scale. For *AVR‐Pik*, which has multiple cognate *R*‐genes both *R* gene frequencies were used as predictors, and the results were visualized as a slice over different frequencies of *Pi‐km*.

### DNA sequence analysis

The *Pyricularia oryzae* ‘70‐15’ v.3.0 (Dean *et al*., 2005) genome assembly and annotation files were downloaded from MycoCosm (Grigoriev *et al*., 2014). All the pathogen (*P. oryzae*) shotgun sequencing samples with collection dates, locations and with ‘rice’ listed as host were downloaded from the NCBI Sequence Read Archive (329 samples). Reads were trimmed using Fastp v.0.23.4 (Chen, 2023) and then aligned to the reference genome sequence using bwa‐mem2 v.2.2.1 (Vasimuddin *et al*., 2019). Alignments were sorted using Samtools v1.20 and then fed through the Bcftools v1.21‐106‐g2d84e07e mpileup‐call pipeline (Danecek *et al*., 2021) to generate joint reference and variant calls at every reference genome position for all resequencing libraries.

The read alignment (binary alignment map) files were reused for separate variant calling only in the *AVR‐Pita1* and *AVR‐Pik* gene regions. Gatk v.4.6.1.0 (McKenna *et al*., 2010) HaplotypeCaller and GenotypeGVCFs were used on each sample independently and then converted into pseudo‐genome assembly sequences using bcftools consensus. Separate variant calling for these two genes was necessary because bcftools did not handle overlapping complex variants well, so we determined each sample needed to be genotyped independently. These sequences were added to the newly sequenced gene sequences described below.

The draft DNA sequence data of the *AVR* genes from the sequencing facility (as outlined above in ‘DNA sequencing of the *AVR* genes’) were assembled with the Vector NTI software (Life Technologies Corp., Carlsbad, CA). DNA sequence assemblies of the ORF were aligned with Clustal Omega (Sievers *et al*., 2011). PCR products of *AVR‐Pi9*, *AVR‐Pizt*, *AVR‐Pib* and *ACE1* were determined to be identical to published sequences. The ORFs of *AVR‐Pita1* and *AVR‐Pik* in isolates consisting of 132 and 151 samples, respectively, were analyzed along with 109 published ORF sequences of *AVR‐Pita1* and 11 *AVR‐Pik* from GenBank resulting in 241 *AVR‐Pita1* and 168 *AVR‐Pik* sequences, respectively. Together with the sequences derived from aligned of short reads to the *Mo* reference genome assembly, *AVR‐Pita1* had 343 global and 125 US sequences, and *AVR‐Pik* had 289 global and 151 US sequences (Dataset S3, S4).

Multiple sequence alignments for the *AVR‐Pita1* and *AVR‐Pik* coding sequences (CDS) and peptide sequences were generated using ClustalW2 v.2.1 (Larkin *et al*., 2007). Phylogenetic trees for the peptide sequences were created using PhyML‐mpi v.3.3.20241207 (Guindon *et al*., 2010). Although included in the analysis, alleles containing early stop codons (i.e. having truncated peptide sequences) were excluded from phylogenetic trees. Trees were plotted using R packages ape v5.8‐1 (Paradis & Schliep, 2019).

Population structure was inferred for the pathogen simple sequence repeat (SSR) data with the ‘find.clusters’ and ‘dapc’ functions in the R package adegenet v.2.1.11 (Jombart, 2008; Jombart & Ahmed, 2011). The membership probabilities were plotted against the presence/absence of the *AVR* genes using a modified version of the code from the R package ggcompoplot (Zamvar *et al*., 2015).

To further integrate the US collection here with publicly available resequencing libraries, we attempted to call SSR genotypes using the same approach as the *AVR* genes. However, we were unable to align the SSR band lengths to those acquired in this study. The results are still included for completeness in the supplement, but they will not be discussed due to the difficulty in interpreting the results (Dataset S5).

## Results

### *AVR* genes in the United States and global *Mo* population over six decades, and population structure of the US population

We first investigated the presence or absence (P/A) of eight *AVR* genes in US *Mo* isolates collected over six decades using *AVR* gene‐specific primers (Tables S1, S2) and using a sequence‐counting approach for publicly available resequencing datasets (Dataset S2).

Analysis of eight putatively neutral SSR markers with Discriminant Analysis of Principal Components (DAPC) revealed five primary subpopulations in the United States (Fig. 1a). We attempted to include the global samples by deriving expected SSR band sizes by indel calls from short reads, but we found it extremely difficult to match band sizes, so they were not included in this analysis. There was no clear separation between *AVR* gene presence/absence and DAPC clusters, suggesting that *AVR* gene gain and loss are not co‐occurring purely within clonal lineages (Fig. 1).

**Fig. 1:**
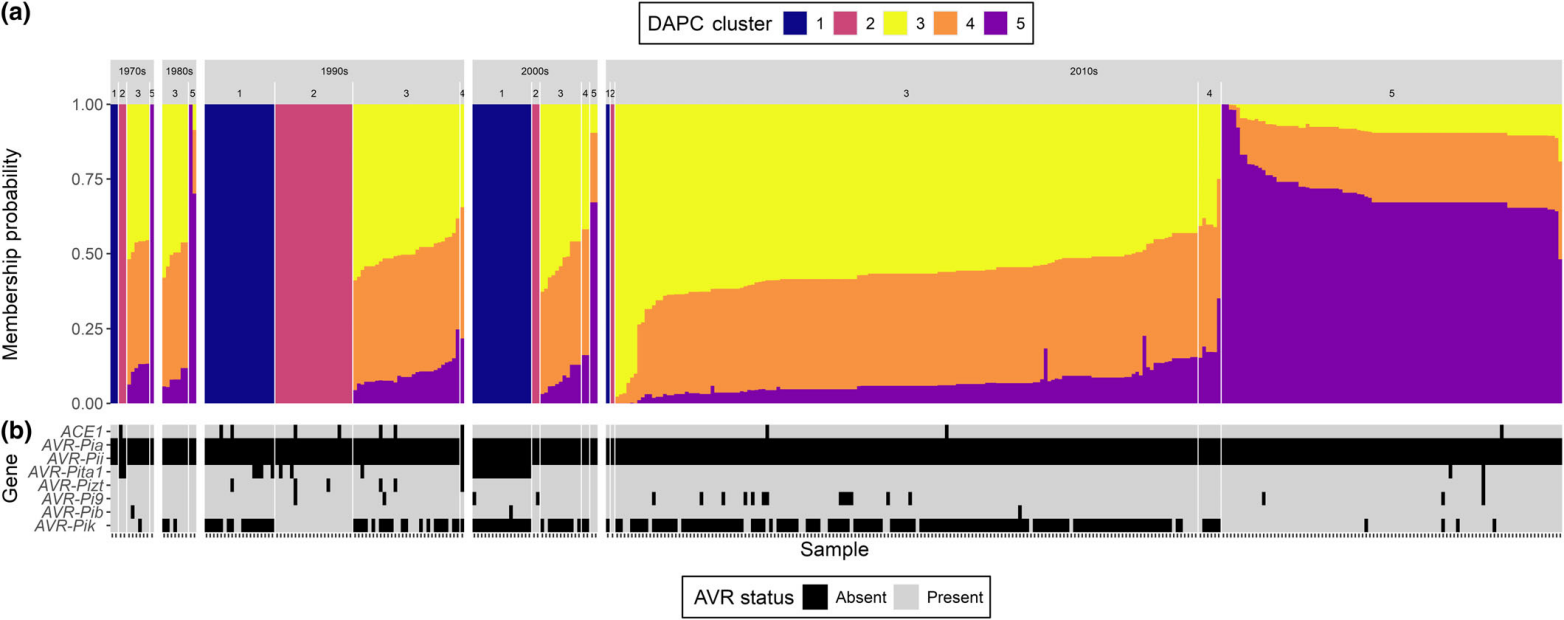
Population structure and avirulence (*AVR*) gene presence/absence of 383 single‐spore isolates from the US *Magnaporthe oryzae* (*Mo*) population. (a) The entire population was grouped into five subpopulations using SSR markers with discriminant analysis of principal components. Pathogen isolates are grouped first by decade of collection and then by cluster assignment. Five colors represent the clusters (populations) inferred by SSR marker data. (b) The *AVR* gene presence/absence status for each isolate is displayed below for each corresponding sample.

In the global *Mo* samples (Figs 2a, 3a), *AVR‐Pik* and *AVR‐Pizt* stayed highly prevalent (> 75%) from the 1960s to the 2010s, and *AVR‐Pia* stayed rare (< 15%). *ACE1* had a small increase in prevalence but was close to 100% over the same time period. *AVR‐Pii* started close to 50% prevalent and has disappeared almost entirely. *AVR‐Pita1* has experienced a slow decline of *c*. 5% per decade, now (as of 2020) *c*. 50% prevalence globally. *AVR‐Pi9* increased from the early 1980s, dipped slightly to 75% in the late 2010s and is now close to 100% prevalence again, and *AVR‐Pib* has remained *c*. 75% prevalence (Fig. S1A).

**Fig. 2:**
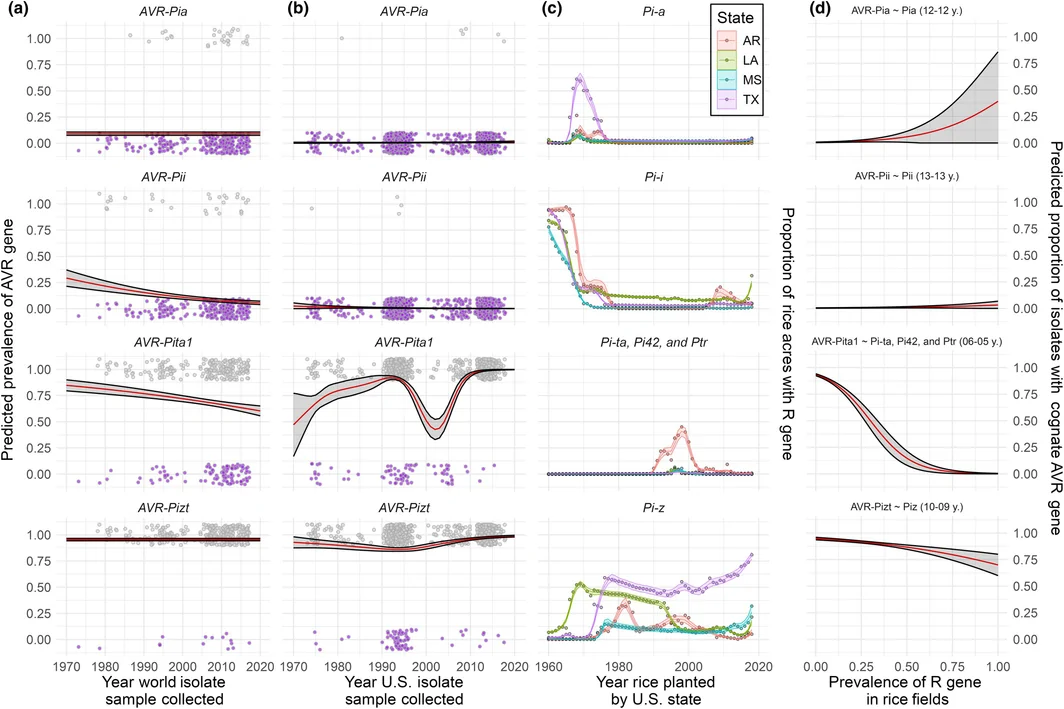
Evolutionary dynamics of avirulence (*AVR*) genes, *ACE1*, *AVR‐Pia*, *AVR‐Pii*, *AVR‐Pita1* and *AVR‐Pizt*, in the global (*n* = 328) and US (*n* = 824) *Magnaporthe oryzae* (*Mo*) populations in response to corresponding cognate rice (*Oryza sativa* L.) *R* genes, *pi‐d*, *Pi‐a*, *Pi‐i*, the ‘*Pi‐ta*, *Pi42* and *Ptr*’ supercluster *and Pi‐z*, frequency changes. Information for corresponding AVR gene and cognate R gene can be found by scanning left to right, except for *ACE1* and *pi‐d*, which are not cognates but still shown for completion. Predicted prevalence of *AVR* genes is shown by year for global (a) and US (b) *Mo* isolates. The gray and purple dots correspond to isolates with and without one of the five *AVR* genes respectively, detected by either resequencing (global) or PCR (b) detection methods, with points jittered to make individual samples more discernible. The red line is the trend line estimated from the binary occurrence data estimated using a spline, and the gray ribbon is the SE for the predictions at each point in time. (c) The proportion of rice acreage in each year and state having cognate *R* genes, with years having no rice varieties with known *R* gene composition filled in using linear interpolation. The colored lines are loess‐smoothed trends for the proportion of US rice acreage having each of the five *R* genes. (d) Model predictions for the effect of changes in rice *R* gene frequency on the pathogen *AVR* gene arsenal, with the red line being the marginal prediction for the *AVR* prevalence at a given *R* gene frequency. Lagged *R* gene frequencies for state of isolate collection were used as the predictor in the model, with the average of some previous number of years given in the strip of text above each facet. The best *R* gene lag to predict the *AVR* gene prevalence at a point in time was identified using an exhaustive search of all continuous ranges of years 0–15 yr before a pathogen isolate collection, with the best *R* gene lag corresponding to the predictor with the lowest *P*‐value in a generalized linear mixed model to predict *AVR* gene prevalence.

**Fig. 3:**
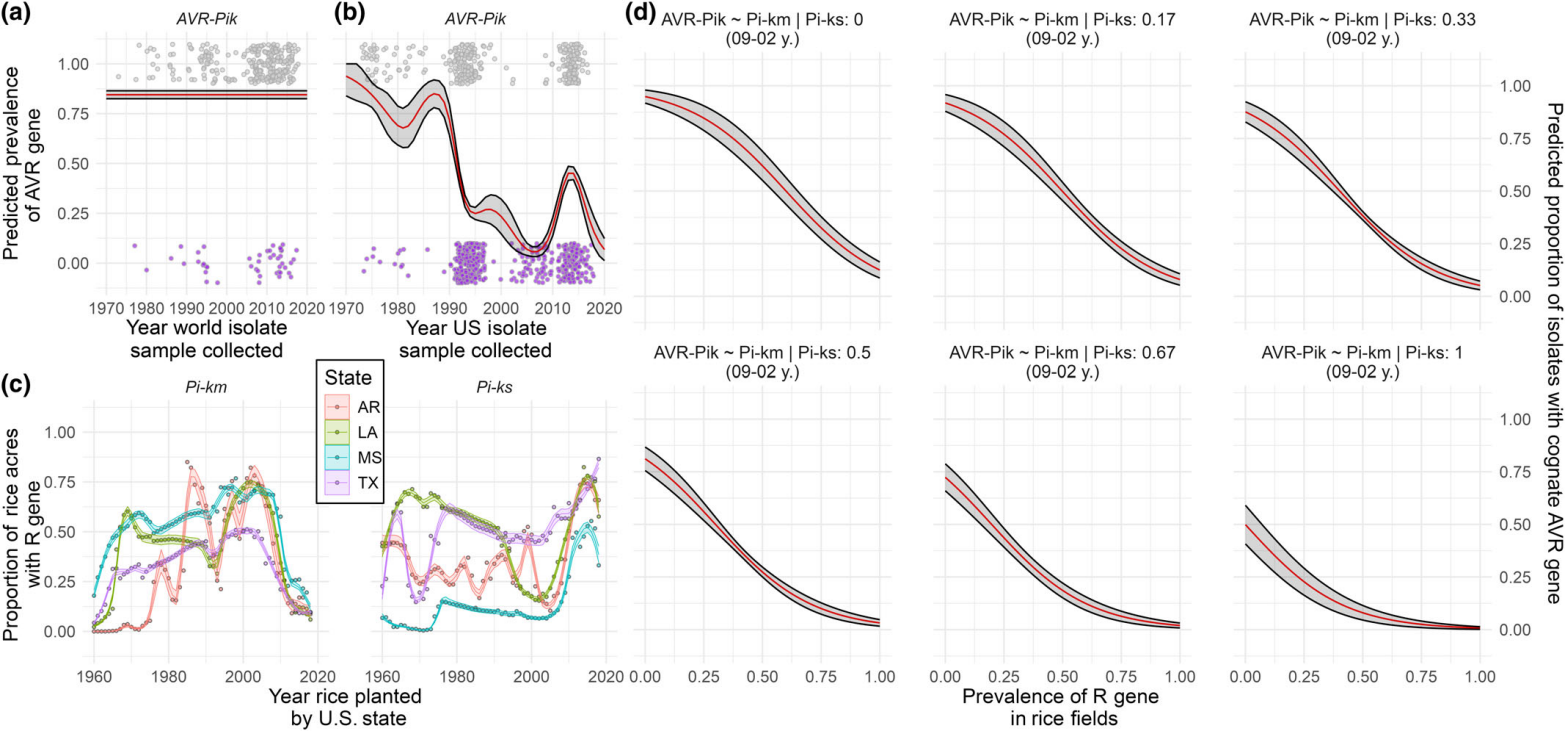
Evolutionary dynamics of avirulence (*AVR*) gene, representing multiple *AVR‐Pik alleles*, in the global (*n* = 328) and US (*n* = 824) *Magnaporthe oryzae* (*Mo*) populations in response to cognate rice (*Oryza sativa* L.) *R* genes, *Pi‐km and Pi‐ks*, frequency changes. Predicted prevalence of *AVR‐Pik* by year for global (a) and US (b) *Mo* isolates. The gray and purple dots correspond to isolates with and without *AVR‐Pik* respectively, detected by either resequencing (global) or PCR (b) detection methods, with points jittered to make individual samples more discernible. The red line is the trend line estimated from the binary occurrence data estimated using a spline, and the gray ribbon is the SE for the predictions at each point in time. (c) The proportion of rice acreage in each year and state having cognate *R* genes *Pi‐km* or *Pi‐ks*, with years having no rice varieties with known *R* gene composition filled in using linear interpolation. The colored lines are loess‐smoothed trends for the proportion of US rice acreage having each of the 2 cognate *R* genes. (d) Model predictions for the effect of changes in rice *R* gene frequency, *Pi‐km*, on the pathogen *AVR* gene arsenal, with the red line being the marginal prediction for the *AVR* prevalence changing *Pi‐km* frequency at fixed *Pi‐ks* frequencies given in the strip title (0, 0.17, 0.33, 0.50, 0.67, and 1). Lagged *R* gene frequencies for state of isolate collection were used as the predictor in the model, with the average of some previous number of years given in the strip of text above each facet. The best lag was chosen using the same method as for the other five *R* genes. There are six panels because the effect of one *R* gene on the *AVR* gene depends on the frequency of the other cognate *R* gene.

The trends for *ACE1* (Fig. S2), *AVR‐Pia* and *AVR‐Pizt* are qualitatively similar between the United States and global *Mo* isolates. Unlike in the global samples where *AVR‐Pii* started off at moderate frequency and trended down, *AVR‐Pii* started off and has remained nearly completely absent in the US population. *AVR‐Pita1* has experienced a different trend in the United States compared to the global samples, experiencing growth until the 1990s followed by a sharp decline to < 50% prevalence in the early 2000s before rebounding to nearly 100% prevalence in the 2010s. *AVR‐Pik* prevalence has been similarly dynamic, detected in > 50% of isolates before 1990 after which point it almost entirely disappeared in the mid‐2000s, bounced back to *c*. 50% in the early 2010s and then crashed down again. *AVR‐Pik* showed essentially no change in the global population. *AVR‐Pi9* has remained highly prevalent in the United States since sampling began, unlike in the World where it has generally trended upward (Fig. S1A,B). The cognate *R* gene, *Pi9*, has not been deployed in the United States, which could explain the different trend for this *AVR* gene.

### Correspondence of deployed *Pi‐ta* and *Pi‐km/Pi‐ks* (*Pi‐k#*) with P/A of *AVR‐Pita1* and *AVR‐Pik*

To determine whether deployed *R* genes influenced the observed molecular changes in the *AVR* genes in the pathogen population, we analyzed deployed *R* genes in the Southern United States since 1960 (Figs 2c, 3c; Dataset S1, S2).

The frequency of rice *R* gene *Pi‐ta* in the US rice fields increased since the late 1980s, reached a peak in the late 1990s and then it decreased until there was almost no deployment in the late 2000s (Fig. 2c). After *Pi‐ta‐*containing varieties were deployed, the frequency of *AVR‐Pita1* in *Mo* was gradually reduced. The *Mo* population shifted to isolates without *AVR‐Pita1* and/or with more AVR‐Pita1 protein variants differing in a few amino acids sequentially after *Pi‐ta* was deployed. After 5–6 yr of *Pi‐ta* maximal deployment in the mid‐1990s, the frequency of *AVR‐Pita1* decreased to the lowest point in the early 2000s. Overall, the subsequent increased frequency of *AVR‐Pita1* coincided with a reduction in *Pi‐ta* in the field. The lowest presence of *AVR‐Pita1* was 5–10 yr after *Pi‐ta* reached its highest peak. Based on modeling of the *AVR‐Pita1* response to changes in *Pi‐ta* frequencies, a background prevalence of the *R* gene *c*. 80% is expected to be reduced to < 10% at 50% *Pi‐ta* frequency, indicating this *AVR* gene is highly sensitive to the potent *Pi‐ta* resistance gene (Fig. 2d).

Many rice varieties grown in the United States since 1960 carry *Pi‐ks*, and since 1965, they carry *Pi‐km* (Table S3). We use *Pik*# to refer to the frequency of *Pi‐km*+*Pi‐ks*. Since *Pi‐ks* and *Pi‐km* have been deployed for a long time, the frequencies of *Pi‐km* and *Pi‐ks* showed multiple peaks over decades resulting in multiple reductions in the frequency of *AVR‐Pik* (Fig. 4a–c). The maximal deployment of *Pi‐km* occurred in the 1980s (Fig. 3c; Table S3). There was a rapid loss of *AVR‐Pik* between the late 1980s and early 1990s after heavy deployment of *Pi‐km*. The same pattern occurred in the early 2000s, when *Pi‐km* frequency reached another peak, the frequency of *AVR‐Pik* decreased to its lowest level in the late 2000s. These findings suggest that *AVR‐Pik* in the pathogen was influenced by *Pi‐km*, about half a decade after *Pi‐km* and *Pi‐ks* were heavily deployed. Further analysis showed that the prevalence of *AVR‐Pik* was best predicted if the average *Pik#* frequency in the preceding −9 to −2 yr was used. A joint model was needed to account for the effects of both *R* genes on the *AVR* gene frequency (Fig. 3d). *Pi‐km* appears to have a weaker impact on the prevalence of *AVR‐Pik* alleles as compared to the corresponding dynamics for *AVR‐Pita1* (Fig. 3d).

**Fig. 4:**
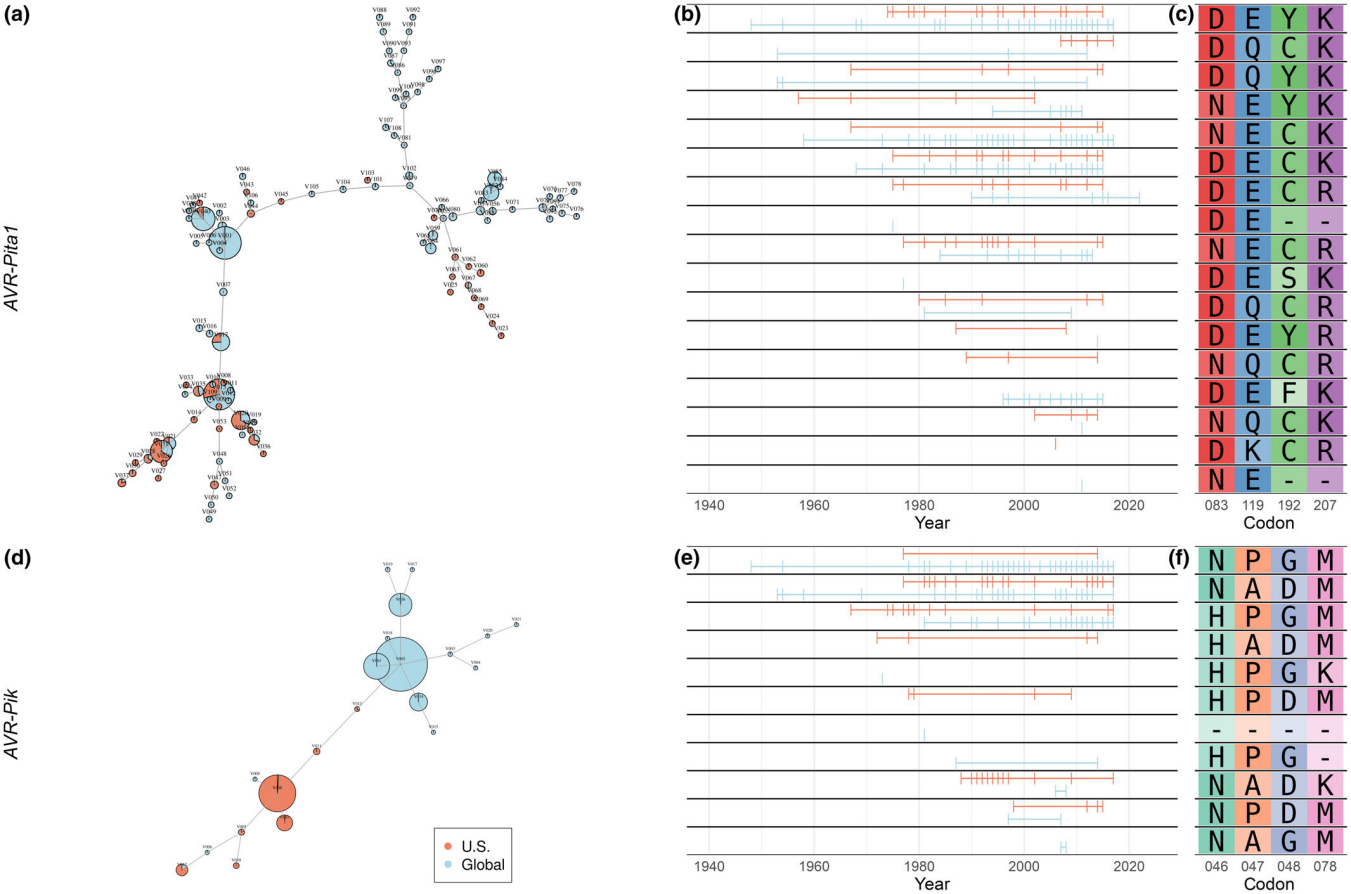
Genealogy and amino acid polymorphism of two avirulence (*AVR*) genes, *AVR‐Pita1* and *AVR‐Pik*, in the *Magnaporthe oryzae* (*Mo*) populations. Haplotype network representing stepwise changes in nucleotide composition of *AVR‐Pita1* (a – *n*_Global_ = 343, *n*_U.S_. = 125) and *AVR‐Pik* (d – *n*_Global_ = 289, *n*_U.S_. = 151). Each labeled variant, such as V001, corresponds to a unique haplotype sequence for that gene. Variants are ordered such that highly related haplotypes are connected by a gray line. The pie chart shows the proportion of individual haplotype sequences coming from the global (light blue) or US (salmon) isolates. Occurrences of each protein haplotype, grouped by four key residues, are also shown in a time‐line view (b and e) along with the corresponding amino acids at these codon positions (c and f). Lines connect the first and last observations of the haplotypes in this study.

*AVR‐Pik* was less efficient to overcome the *R* gene's selection pressure compared to *AVR‐Pita1*, suggesting that *AVR‐Pik* is dispensable for *Mo* (Figs 2d, 3d). Considering the overall low frequency of *AVR‐Pik* (compared to *AVR‐Pita*, *AVR‐Pi9*, *AVR‐Pizt*, *AVR‐Pib* and *ACE1*), heavy deployment of *Pikm/Pikh* over time negated any benefit.

### Evolutionary relationships among nucleotide variants of *AVR‐Pita1* and *AVR‐Pik*

There were 109 nucleotide haplotypes for *AVR‐Pita1* from 26 segregating sites, and 21 haplotypes for *AVR‐Pik* from 12 segregating sites (Datasets S3, S4; Fig. 4a/d). There was some bias in the representation of clonal lineages for *AVR‐Pik*, with the large group represented by variant V002 having only representation in the global samples but the group represented by V008 having nearly only representation in the US samples. The picture was much less clear for *AVR‐Pita1*, with one lineage related to V102 and V080 only being represented in the global samples set, and lineages related to V061 and V014 being strongly over‐represented in the US set.

We highlighted four critical amino acid residues (see Takahashi *et al*., 2010, for *AVR‐Pita1*; Maqbool *et al*., 2015, and Li *et al*., 2019, for *AVR‐Pik*) for each of the AVR proteins and noted each time we observed them in the United States and global sample sets (Fig. 4b/c,e/f). For the most part, once new variants of AVR‐Pita1 or AVR‐Pik were observed, they stayed around for the duration of sampling. The variant of AVR‐Pita1 that was pathogenic to resistance protein Pita1, NQCR, was first detected in the United States in the late 1980s, after which point one more derivative variant was observed only in the United States – NQCK in the early 2000s. Two AVR‐Pik variants without known cognate Pi‐k allelic variants, HADM and NADK, were found much more commonly in the United States than in the World samples (4/4 and 12/14, respectively), where *Pi‐k#* has been widely deployed.

### Validation of interaction specificity of *AVR* genes with their cognate *R* genes

Gene for gene theory predicts that major blast *R* genes are effective in preventing the infections of *Mo* strains with their corresponding *AVR* genes. To determine the specificity of interaction between *AVR‐Pita1* and *Pi‐ta*, rice varieties Katy and M202 with and without the *Pi‐ta* gene were challenged with one isolate carrying *AVR‐Pita* IB49 (Fig. 5). Rice variety M202 lacks *Pi‐ta* and was susceptible to all *AVR‐Pita1* haplotypes as expected. Katy with *Pi‐ta* was resistant to all *AVR‐Pita1* haplotypes.

**Fig. 5:**
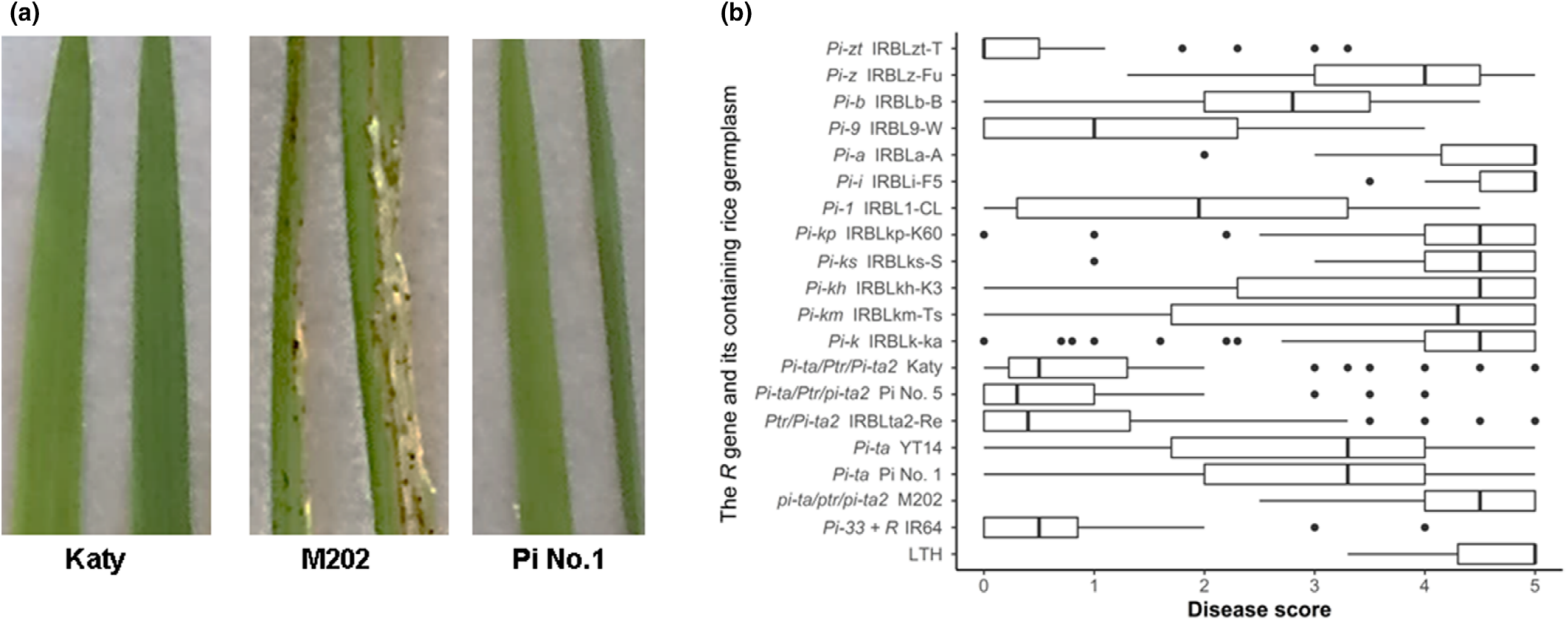
Validation of the efficacy of an avirulence (*AVR*) gene as an indicator for the resistance reaction of its cognate rice (*Oryza sativa* L.) *R* gene. (a) Blast disease symptoms of the representative rice varieties. The *Magnaporthe oryzae* (*Mo*) race IB49 (isolate ML1_2014) was used for inoculation. The images are shown only to demonstrate the range of symptoms in the experiment. (b) Distribution of disease scores. The *X* axis indicates scores of disease reactions where 0–2 represents resistance and 3–5 susceptibility, respectively. The *Y* axis indicates IRRI monogenic lines and *R* gene differential lines and the susceptible control LTH. A total of 64 blast strains (Supporting Information Dataset S6) with different *AVR* genes were used to inoculate indicated rice genotypes. The thick line is the median disease score, and the boxes are the 1st and 3rd quartiles. Whiskers extend to the further point within 1.5 times the interquartile range. Points outside this range are marked with dots.

To determine the specificity of interaction between *AVR‐Pik* and *Pik#*, the IRRI monogenic lines carrying *Pi‐1*, *Pi‐km*, *Pi‐kh*, *Pi‐kp* and *Pik* were challenged with the isolates carrying different AVR‐Pik variants. The isolate carrying AVR‐Pik D showed moderate AVR activity with *R* gene *Pi‐km* and *Pi‐kh* (Fig. 5).

To determine whether *AVR* genes are good indicators for the efficacy of their cognate *R* genes for blast control, we selected 64 isolates with different *AVR* genes from recent blast collections and inoculated them onto IRRI monogenic rice lines carrying cognate *R* genes and differential rice lines (Fig. 5; Dataset S4). We showed that line IRBLzt‐T carrying *Pi‐zt* was resistant to 96.38% isolates, line Pi‐no.5 (*Pi‐ta*) was resistant to 93.75% of isolates, cultivar Katy carrying *Pi‐ta* and *Pi‐ks* was resistant to 87.5% of isolates, and line IRBLb‐B carrying *Pi‐9* was resistant to 81.25% of isolates. The corresponding *AVR* genes, *AVR‐Pizt*, *AVR‐Pita1* and *AVR‐Pi9*, were found with high frequency in the field. *Pi‐km* and *Pi‐ks* have been used for six decades by US rice breeders. In recent years, *Pi‐km* was still resistant to 35% of isolates and *Pi‐ks* was only resistant to 1.69% of isolates. As expected, due to lack of *AVR‐Pii* and *AVR‐Pia* in the *M. oryzae* field population, few isolates were incompatible with monogenetic lines carrying *Pii* and *Pia*.

## Discussion

The majority of rice grown in the United States is of domestic origin with many different varieties developed through breeding programs and selection over the years to meet the specific needs of US growers and consumers. These *M. oryzae* isolates collected over a 56‐yr period from commercial rice fields are ideal to investigate evolution and adaptation of a pathogen concurrent to changes in resistance genes deployed in host plants. We provided evidence that changes in *R* gene composition in rice were followed by genetic change in the *M. oryzae*, with changes taking 5–10 yr depending on the *AVR‐R* pair; these changes were quite different from the patterns seen in global samples (Figs 2a/b, 3a/b). Note that different methods were used to detect the *AVR* genes in the United States (PCR) and global (primer counting) datasets, so there is a possibility some of the difference seen between the two sets is due to a difference in methodology. Follow‐up studies using whole genome sequencing on the US panel will clarify these potential differences. Regardless, there were both changes in *AVR* presence/absence and haplotype diversity.

The most stark changes were apparent in *AVR‐Pita1* and *AVR‐Pik*. In both cases, increase in the *R* gene prevalence corresponded to a decreased frequency of the cognate *AVR* gene 5–10 yr after broad *R* gene deployment. The frequency of *AVR‐Pik* has been increasing again with declining *Pi‐km* deployment. Additionally, *AVR‐Pia* and *AVR‐Pii*, corresponding to *R* genes that were widely deployed in the 1960s and the early 1970s (Dataset S2), were very rare in the US *Mo* population. Widespread presence and sequence conservation of *AVR‐Pib*, *AVR‐Pi9*, *AVR‐Pizt* and *ACE1* through the decades is consistent with absence of their cognate *R* genes. This result suggests that the *R* genes *Pi‐b*, *Pi9*, *Pizt* and *Pi33* can be stacked to provide durable and effective blast resistance (Mundt, 2018). Additionally, further follow up work would help clarify why some *AVR* genes, such as *AVR‐Pizt*, seem to respond very slowly over a long time period, despite significant deployment of the cognate *R* gene *Pi‐z*. We note that the limitations of our findings regarding *AVR* presence/absence are limited by the extent and availability of pathogen survey samples during the period of sample collection and recommend future sampling experiment sample as deeply as possible to capture low‐frequency, standing variation in the pathogen.

A pathogen fitness penalty may result from mutation of an *AVR* gene due to loss of avirulence, which is necessary to overcome an *R* gene. This fitness penalty in the pathogen is hypothesized to correspond to durable resistance for that *R* gene (Leach *et al*., 2001). Losses of AVR causing lower spore productions have been reported in rust pathogen *Melampsora lini* (Thrall & Burdon, 2003), oilseed rape phoma stem canker pathogen *Leptosphaeria maculans* (Huang *et al*., 2010) and potato late blight pathogen *Phytophthora infestans* (Montarry *et al*., 2010). In the rice blast fungus, reduced aggressiveness associated with loss of any particular *AVR* gene has been recently implied in laboratory studies (Gao *et al*., 2021). The fitness cost imposed by *AVR* gene mutations can also be determined whether there is persistence or decline in frequency of a particular virulence gene (mutated *AVR* gene) when the corresponding *R* gene is gone from the field (Bahri *et al*., 2009). Additionally, the population rapidly regained an *AVR‐Pita1* gene after the *Pita/Pi42/Ptr* region (Jia *et al*., 2012) was removed from the field (Fig. 2b/c). The presence of *AVR‐Pik* is correlated with levels of *Pi‐km* in the field, with *AVR‐Pik* levels rising again as the *R* gene levels decrease (Fig. 4a). These results suggest that the virulence alleles of these *AVR* genes might impose fitness costs on the pathogen. However, more direct, nonsurvey measures of pathogen fitness from laboratory or field experiments would clarify which level at which *AVR* gene loss impacts the pathogen life cycle.

The continuing absence of *AVR‐Pia* and *AVR‐Pii* after *Pi‐a* and *Pi‐i* were not deployed in the field suggests that there is not a fitness penalty associated with total loss of these *AVR* genes, or that near‐total loss prevented the gene frequency from returning. If only virulence alleles of *AVR* genes in pathogen carry fitness penalties, the frequencies of AVR alleles of *AVR* should be increased. However, some rare avirulent haplotypes of *AVR‐Pita1* only appeared in a short time span, even after almost no *Pi‐ta* was deployed in the field. For example, some haplotypes were only present once in the *Mo* population in the early 2010s when the frequency of *Pi‐ta* was only 7.5% in the field. The results support the idea that *AVR‐Pita1* gene mutation to new *AVR* alleles also carries a fitness penalty, which can be observed by a reduction in pathogen fitness on the host without the *R* gene (Leach *et al*., 2001). Widespread deletion and sequence variation of *AVR‐Pita* was not a surprise because this *AVR* gene is a member of a subtelomeric gene family and thus they are easier to lose through evolution (Orbach *et al*., 2000; Khang *et al*., 2008; Chuma *et al*., 2011). In addition to variation in amino acid sequence and presence/absence polymorphism in different strains, *AVR‐Pita1* shows variable location at subtelomeric locations on different core chromosomes in different field isolates (Chuma *et al*., 2011). Specifically, avirulent *AVR‐Pita* alleles are located near the ends of Chromosomes 1, 3, 4, 5, 6, 7 or in supernumerary chromosomes in different rice‐infecting isolates and other host‐specific strains. This gene movement, termed multiple translocations, is suggested to be a mechanism for the fungus to regain deleted *AVR* genes, although at different chromosomal locations. Transposable elements, shown to directly contribute to emergence of virulent *AVR‐Pita* isolates (Zhou *et al*., 2007; Singh *et al*., 2014), are also implicated in multiple translocations of *AVR‐Pita1* (Chuma *et al*., 2011). Understanding the molecular mechanisms underpinning *AVR* gene dynamics and structural variability should be an important focus for future research.

Once new *R* genes are deployed, pathogen strains with their cognate *AVR* genes are short lived in commercial fields. Generally, such pathogens either impose nucleotide variation or deletion of *AVR* genes to overcome barriers caused by *R* genes in the host. Once an *R* gene in the host decreases or disappears, the pathogens benefit from the cognate *AVR* gene. One earlier study suggests that the host *R* gene influenced the deletion of the *AVR* genes. Xing *et al*. collected 28 *M. oryzae* field isolates from the *Pita*‐containing cultivars and 75 isolates from the non‐*Pita*‐containing cultivars. They identified eight and six virulent isolates to Katy (*Pi‐ta* containing cultivar) from 28 and 75 field isolates, respectively. A chi‐square test showed an association of virulent isolates with the *Pi‐ta* gene (Xing *et al*., 2013).

Co‐evolution between *Pi‐k*# and *AVR‐Pik* is a classic example of an ‘arms race’, which is interpreted as *Pi‐k*# having developed multiple resistant alleles gradually to deal with newly evolved *AVR‐Pik* genes (Kanzaki *et al*., 2012; Wu *et al*., 2014). *Pi‐km* and *Pi‐ks* have been used for over four decades in the Southern United States and are still used intensively in breeding programs (Anna McClung, pers. commun.). *Pi‐km* has a broader disease spectrum than that of *Pi‐ks*. Detailed analysis of *AVR‐Magk1* will help to understand the resistance stability of *Pi‐ks* (Sugihara *et al*., 2023). Nevertheless, even though there appears to be some fitness benefit associated with *AVR‐Pik*, heavy deployment of *Pi‐km/Pi‐kh* has already negated the beneficial effect of *AVR‐Pik* and suggests that *Pi‐km/kh* may not be effective in preventing rice blast infection in the future in the Southern United States.

In contrast to *Pi‐k*#, co‐evolution between *Pi‐ta* and *AVR‐Pita1* follows ‘trench warfare’, based on one *Pi‐ta* resistance allele fighting with fast evolving *AVR‐Pita1*. In the Southern United States, *Pi‐ta* occurs in a large linkage block on Chromosome 12, which was bred into a dozen rice varieties in the United States (Zhao *et al*., 2018). The linkage block including *Pi‐ta*, *Pi42* and *Ptr* has existed in almost all *Pi‐ta* containing cultivars in the United States. *Ptr* also confers broader spectrum blast disease resistance than *Pi‐ta* (Jia *et al*., 2012; Zhao *et al*., 2018). However, *Pi‐ta* containing rice varieties thus far have not been competitive in yield to those without it. Consequently, *Pi‐ta* containing rice varieties were not widely deployed (Fig. 2c), because genetic components related to low yield are in the same linkage block (Wang *et al*., 2015). Hence, most *Mo* isolates in the United States still carry *AVR‐Pita1* suggesting that there exists an opportunity for developing high‐yielding rice varieties with the *Pi‐ta* gene.

Analysis of 886 isolates of rice blast fungus from 152 sites in 51 countries including 76 US isolates revealed three lineages and one recombining group (Thierry *et al*., 2022). The US isolates examined were in the clonal lineage group 2. We previously demonstrated that the US blast was under asexual reproduction and is remarkably diverse relative to world‐wide population with most variation happening over time (X. Wang *et al*., 2017). We did not detect correspondence between P/A markers of *AVR* genes and SSR markers (Fig. 1a/b; Dataset S7). Therefore, we cannot determine whether or when selection against any *AVR* genes may have also influenced the clonally linked genome. Previously, phylogenetic correlations with P/A of the effector genes of *M. oryzae* were demonstrated using high‐density genetic markers and many more effector genes (Latorre *et al*., 2020; Thierry *et al*., 2022). Lack of correspondence in our study may be explained by limited numbers of SSR markers and *AVR* genes used. Further analysis of whole genome sequences of our US blast isolates will help understand the molecular mechanism of origin and evolution of rice blast fungus.

Rice blast disease is the most disastrous disease for rice production globally. *Magnaporthe oryzae* is a highly unstable fungus showing rapid changes in *AVR* genes. This study uncovered a sophisticated co‐evolutionary mechanism of *R* genes in rice and *AVR* genes in *M. oryzae*. We showed that *M. oryzae* strains undergo mutations and even total deletion of *AVR* genes to overcome the pressure imposed by the corresponding *R* gene. The most recent field isolates have *AVR‐Pi9*, *ACE1*, *AVR‐Pib*, *AVR‐Pizt* and *AVR‐Pita1*, suggesting that the corresponding *R* genes, *Pi‐9*, *Pi‐b*, *Pi‐33*, *Pi‐zt* and *Pi‐ta* are still effective in the United States. Our analysis of the genetic changes in *AVR* genes in 840 isolates from commercial rice fields in the United States over 58 yr suggests that *R* gene stacking and recycling can be used to achieve the durability of blast resistance. The information obtained here is useful to understand how to develop effective strategies to manage rice blast disease in commercial fields of the Southern United States.

Our study shows the value of tracking of *AVR* gene dynamics relative to the complement of *R* genes deployed in farmers' fields regionally. Rapid recovery of *AVR‐Pita* in the Southern US population after removal of *Pi‐ta* from the field suggests that this *AVR* gene may provide a field fitness advantage on rice lacking this *R* gene. Therefore, efforts to separate *Pita*‐mediated resistance from its associated yield penalty in US germplasm would be beneficial. By contrast, *AVR‐Pita* occurs at low levels in other pathogen populations (Selisana *et al*., 2017), indicating that each population has been shaped by its own local history. For the population we studied, lack of diversity in *AVR‐Pi9*, *ACE1*, *AVR‐Pib* and *AVR‐Pizt* is correlated with lack of deployment of their corresponding *R* genes. Although these *R* genes would currently be useful for combating the Southern US pathogen population, strategic deployment and continuous tracking of *AVR* gene dynamics are warranted. While we found evidence to potentially suggest the US population is quite isolated from world‐wide populations as seen with the differing pattern of AVR gene frequencies, additional studies resequencing the US samples would be necessary to further investigate the relationships.

This study provides some evidence for management of rice blast in the future. We suggest that continuous tracking of *AVR* loss and recovery in regional populations provides the best insight into potential population level fitness for that particular *AVR* gene, which is a characteristic associated with predicting durable resistance genes for blast disease. Diverse sampling should be undertaken with enough density to track the relationship between the pathogen‐host deployed resistance genes and can show when it is likely that pathogens are overcoming the resistance gene and how quickly an additional allele may be necessary to be deployed.

## Competing interests

None declared.

## Author contributions

YJ, YW and BV designed research. XW, YJ, MHJ, YH and YW performed research. XW, GTB, AMH‐K, QDR and YJ analyzed data. XW, GTB, AMH‐K, YJ and BV wrote the paper. QDR, MHJ, YH and YW edited and approved the manuscript. XW and GTB contributed equally to this work.

## Disclaimer

The New Phytologist Foundation remains neutral with regard to jurisdictional claims in maps and in any institutional affiliations.

## Supporting information


**Dataset S1** AVR gene presence absence determined from public resequencing data and from the rapid identification of AVR genes in this study – source data for Figs 2(a,b) and 3(a,b). Sample IDs have been provided with the NCBI sample (SRR) and project (PRJNA) identifies.
**Dataset S2** Interpolated values for rice R gene prevalence by US state and year – source data for Figs 2(c) and 3(c). The interpolated values were calculated using linearization when year‐state combinations were missing.
**Dataset S3** Nucleotide polymorphisms in AVR‐Pita1 in this study – source data for Fig. 4(a), given in nucleotide coordinates in the coding sequence.
**Dataset S4** Nucleotide polymorphisms in *AVR‐Pik* in this study – source data for Fig. 4(b), given in nucleotide coordinates in the coding sequence.
**Dataset S5** Predicted SSR fragment sizes for public *Mo* resequencing libraries.
**Dataset S6** Pathogenicity assay of *Magnaporthe oryzae* isolates on the monogenetic lines with indicated blast resistance genes – source data for Fig. 5.
**Dataset S7** Single‐spore field isolates of *Magnaporthe oryzae* used in this study and presence/absence of each *AVR* gene – source data for Fig. 1.

**Fig. S1** Evolutionary dynamics of AVR genes *AVR‐Pi9* and *AVR‐Pib*, with cognate rice *R* gene *Pi‐b*.

**Fig. S2** Changes in the frequency of non‐cognate AVR gene *ACE1* and *R* gene *Pi‐d*.

**Methods S1** Purification, isolation and storage of *Magnaporthe oryzae* samples.
**Methods S2** Gene specific primers for detection of AVR genes in *Magnaporthe oryzae* isolates.
**Methods S3** Fungal DNA extraction, PCR amplification and purification.
**Methods S4** Plant growth and pathogenicity assays for variety challenge with *Magnaporthe oryzae* isolates.

**Table S1** Single spore isolate of *Magnaporthe oryzae* along with sequencing identifiers, year and state from which sample was collected, race identifier if available, and presence/absence of eight AVR genes.
**Table S2** Primers used for detecting or sequencing the AVR genes.
**Table S3** Rice acreage by variety, year and US state.
**Table S4** R gene status for rice varieties grown in the United States during the study period.
Please note: Wiley is not responsible for the content or functionality of any Supporting Information supplied by the authors. Any queries (other than missing material) should be directed to the *New Phytologist* Central Office.

## Data Availability

All code necessary to reproduce the figures and statistics presented in this study are available at the following GitHub repository: https://github.com/USDA‐ARS‐GBRU/Rice_PathogenDefenseArsenal. Variant calling data for the 329 NCBI SRA entries are available at doi: 10.15482/USDA.ADC/28850993.v1. *AVR‐Pia* sequence is available in GenBank accession no.: AB498873.1. Public sequencing data were sourced from the following NCBI SRA projects: PRJDB14584, PRJDB15332, PRJDB4326, PRJNA304094, PRJNA324432, PRJNA354675, PRJNA354811, PRJNA376429, PRJNA391416, PRJNA417903, PRJNA438312, PRJNA577277, PRJNA670311, PRJNA748547, PRJNA826999, PRJNA866295, PRJNA881958 and PRJNA973860.
