# Supplementary material for: Avirulence genes of Magnaporthe oryzae are a pathogen defense arsenal shaped by cognate rice resistance genes in the field
Source: New Phytol. 2026 Oct 12;252(5):2043–55. doi: 10.1111/nph.71586 (PMC13738682; doi:10.1111/nph.71586)
Supplement: Supplementary file 1 [file NPH-252-2043-s003.docx]

New Phytologist Supporting Information

Article title: Avirulence genes of *Magnaporthe oryzae* are a pathogen defense arsenal shaped by cognate rice resistance genes in the field

Authors: Xueyan Wang, Grant T. Billings, Amanda M. Hulse-Kemp, Quentin D. Read, Yulin Jia, Melissa H. Jia, Yixiao Huang, Yeshi Wamishe, and Barbara Valent

10 Article acceptance date: 24 August 2026

**Supplemental Methods**

**S1 Purification, isolation, and storage of *M. oryzae***

The rice leaves or panicles with the symptoms of blast disease were sampled from rice fields. The diseased plant tissues were placed in petri dishes on top of the prewetted filter papers for 24 h under light for producing spores from mycelia at room temperature (RT, 24 ℃). A small piece of water agar (approximately 2 cm2) was used to touch the surface of leaves with fungal spores under a dissecting microscope. The spores attached to the water agar were sub-cultured in a water agar medium for 3-5 d at RT under continuous light. The hyphal tips approximately 2 mm2 were isolated from the edge of mycelia containing water agar and transferred to an oatmeal medium plate for 5-7 d in the fungal incubator at 22℃ (Percival, Perry, IA). To isolate a single spore, spores from each purified sample were suspended with sterilized deionized water and uniformly distributed on water agar plate for 24 h under continuous lights at room temperature. Single spores were picked along with water agar under the microscope, and were placed on the surface of oatmeal plate for 5-7 d in the fungal incubator at 22 °C. Sporulated mycelia approximately 2 mm2 derived from a single spore were tipped onto a sterilized filter paper (7cm, Cat No, 1001-070, Whatman, Pittsburgh, PA) over oatmeal media for growth until grey spores occupied the entire filter paper in the fungal incubator. The filter paper with sporulated mycelia and spores was removed into a sterilized petri dish and set in a drier with desiccators until the filter paper completely dried. The desiccated filter papers with mycelia and spores were cut into small pieces (0.5cm × 0.5cm) and stored in sterilized glass jars at -20 °C.

**S2 Gene specific primers**

Two sets of primers for each AVR gene were used for either PCR detection or sequencing. For PCR detection, the sizes of PCR amplicons ranged from 341 to 868 bp. For sequencing, the full length of each gene including partial sequences of the 5’ leader and 3’ trailer was amplified. The published primers were initially used. If these primers failed to amplify, new primers from the conservative regions in the exons or 3 and 5 untranslated regions of the genes were designed (**Table S1**).

**S3 Fungal DNA extraction, PCR amplification and purification**

M. oryzae DNA was directly isolated from desiccated filters (Jia et al., 2014). Specifically, DNA was diluted into ddH2O in ratio of 1:4 for PCR. The PCR mixture for a 96-well plate contained: 989 µL home-made cocktail buffer, 43 µL polyvinylpyrrolidone, 50 µL bovine serum albumin, 43 µL each primer (10 µM) and 21 µL Taq polymerase. The final PCR amplification per reaction is 25 µL consisting of 13 µL ddH2O, 10 µL PCR mixture, and 2 µL DNA of each sample. The PCR program was as follows: 95 °C for 5 mins; 95 °C for 30 s; 57- 63 °C for 30 s; 72 °C for 1 min; repeat step 2 to 4 for 34 times followed by 72 °C for 5 mins. A total of 840 samples were used for analysis (Dataset S1). For AVR gene detection, two technical repeats were performed. Samples without a PCR amplicon after 2 repeats were retested using the sequencing primers to ensure that the absence of amplification was not due to sequence variation at the primer sites. The PCR products were separated into a 1% Tris-acetate-EDTA (TAE) agarose gel, and visualized by a BioRad ChemiDoc MP (BioRad, Hercules, CA).

PCR products were purified with the ExoSAP method following the manufacturer’s instructions. Specifically, for each 96-plate, the following chemicals were mixed in a 1.5 µL tube: 22 µL 10 X AP buffer, 22µL Exonuclease I (NEB #M0293L), 22 µL Antarctic Phosphatase (NEB #M0289L), and 374 µL distilled deionized (dd) H2O. Four µL mixture and 4 µL PCR products were used for each reaction. The plate was incubated at 37 °C for 15 min, 80°C for 15 min and held at 15°C in a PCR machine and then was vacuum dried at room temperature and sent out for sequencing. DNA was sequenced at the USDA-ARS MSA Genomics Laboratory at Stoneville, MS, using Big Dye version 3.1 Cycle Sequencing chemistry and an ABI 3730XL DNA Analyzer using 0.5 ll BigDye v3.1.

**S4 Plant growth and pathogenicity assays**

Each plant accession was grown in an insert with 5-8 seedlings at 25 ℃ with 8-h light and 16-h dark in a greenhouse as described (Chen et al., 2019). M. oryzae isolates were grown in the fungal chamber at 22℃, and 39% relative humidity. At the 3-4 leaf stage, rice seedlings were inoculated with the fungal suspension as detailed (Wang et al., 2015). The 0-5 rating system was used to evaluate disease reaction whereas 0 to 2 as resistant and 3-5 susceptible, respectively. Race of each isolate was identified using the international race differential system (Atkins et al., 1967).
